# In vivo evidence for extracellular DNA trap formation

**DOI:** 10.1038/s41419-020-2497-x

**Published:** 2020-04-30

**Authors:** Shida Yousefi, Dagmar Simon, Darko Stojkov, Antonina Karsonova, Alexander Karaulov, Hans-Uwe Simon

**Affiliations:** 1 Institute of Pharmacology, University of Bern, Bern, Switzerland; 2 Department of Dermatology, Inselspital, Bern University Hospital, University of Bern, Bern, Switzerland; 3 Department of Clinical Immunology and Allergology, Sechenov University, Moscow, Russia

**Keywords:** Cell signalling, Inflammation

## Abstract

Extracellular DNA trap formation is a cellular function of neutrophils, eosinophils, and basophils that facilitates the immobilization and killing of invading microorganisms in the extracellular milieu. To form extracellular traps, granulocytes release a scaffold consisting of mitochondrial DNA in association with granule proteins. As we understand more about the molecular mechanism for the formation of extracellular DNA traps, the in vivo function of this phenomenon under pathological conditions remains an enigma. In this article, we critically review the literature to summarize the evidence for extracellular DNA trap formation under in vivo conditions. Extracellular DNA traps have not only been detected in infectious diseases but also in chronic inflammatory diseases, as well as in cancer. While on the one hand, extracellular DNA traps clearly exhibit an important function in host defense, it appears that they can also contribute to the maintenance of inflammation and metastasis, suggesting that they may represent an interesting drug target for such pathological conditions.

## Facts

- The demonstration of extracellular DNA traps in vivo requires sections of affected tissues, which are to be investigated with special staining techniques. These structures are seen in multiple inflammatory and cancer diseases.
- Measurements of cell-free (cf) DNA either alone or as complexes with granules or other cationic proteins, do not prove the in vivo presence of extracellular traps.
- Although neutrophil extracellular traps (NETs) contribute to pathogen clearance, excessive NET formation promotes inflammation and tissue damage.
- There is experimental evidence that NETs can contribute to metastasis.
- Eosinophil extracellular traps (EETs) can bind and kill bacteria. They are often seen in areas of epithelial barrier defects.

## Open questions

- Under which pathological conditions are extracellular DNA traps suitable drug targets?
- Is there a simple biomarker that reflects extracellular DNA trap formation?
- What is the contribution of extracellular microbe killing compared to intracellular killing following phagocytosis?
- The mechanism of DNA trap formation is unknown.

## Introduction

Since their discovery, neutrophil extracellular traps (NETs) have been implicated as playing a role in host defense since they can disarm and kill bacteria extracellularly^1^. Studies aiming at unraveling the underlying mechanisms of NET formation demonstrated a requirement for the production of reactive oxygen species (ROS)^2–5^. ROS induces actin and tubulin glutathionylation, which is tightly regulated by glutaredoxin 1 (Grx1), an enzyme required for deglutathionylation of actin and microtubulin. Thus, an intact cytoskeleton is required for the formation of NETs^6^. Moreover, optic atrophy 1 (OPA1), one of five GTPase dynamin family members, known to play a role in mitochondrial (mt) fusion, has recently been shown to be required for ATP production through glycolysis in neutrophils. If increases in ATP production are blocked, the assembly of the microtubule network and thus the formation of NETs do not occur^7^. ATP and ATP channel pannexin 1 (Panx1) contribute to NET formation and may represent therapeutic targets^8^.

**Fig. 1:**
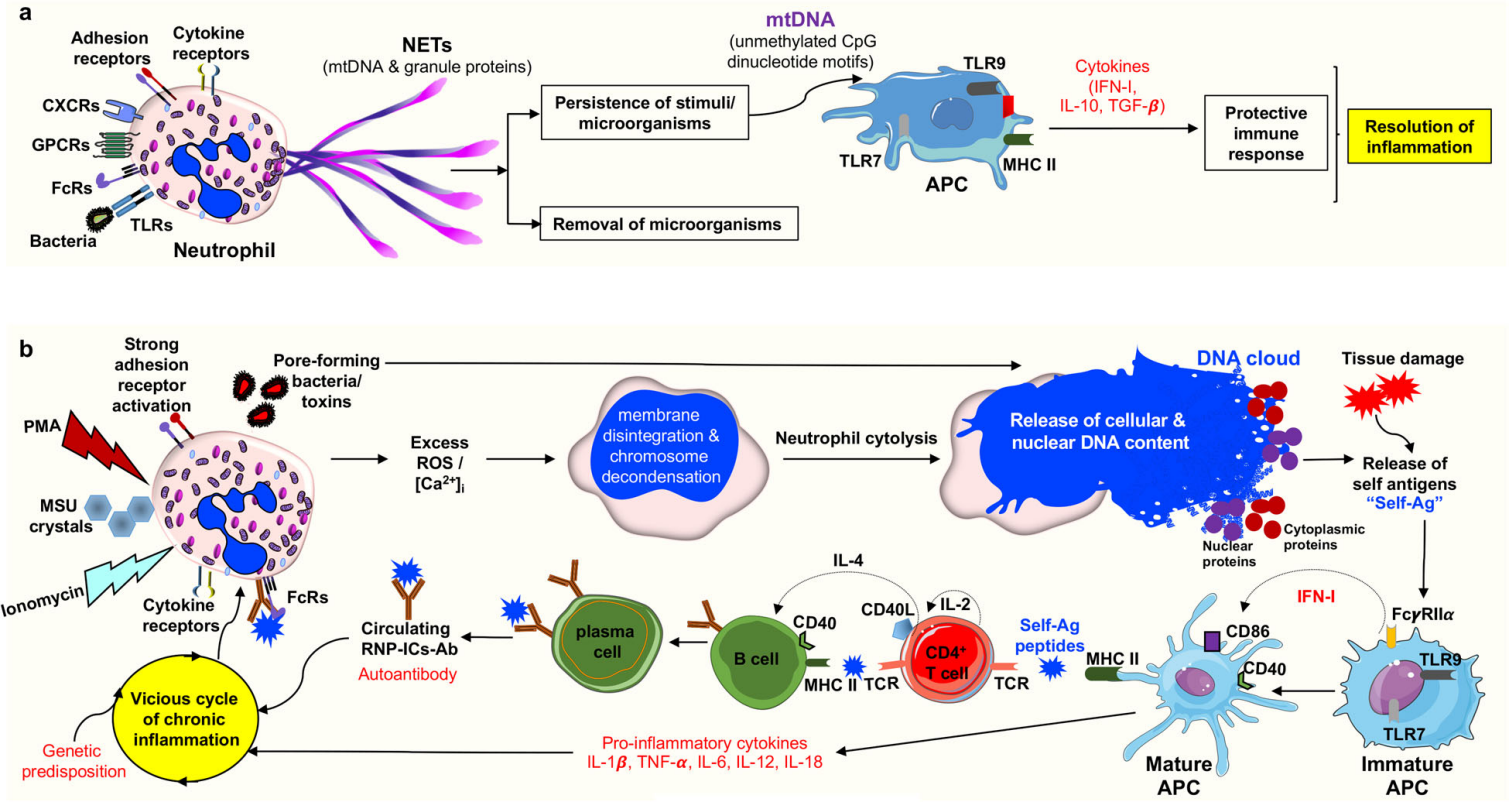
Modulation of the immune system by neutrophil extracellular trap (NET) formation and cytolysis. **a** NET formation: In response to physiological stimuli or bacterial infection, viable neutrophils generate NETs in a NADPH oxidase-dependent manner. NETs consist of a mitochondrial (mt)DNA scaffold, which binds neutrophil granule proteins such as neutrophil elastase (NE) and myeloperoxidase (MPO)^4,6,7,144,152^. If the source of infection/stimulation persists, the released mtDNA, having similarity to viral and bacterial DNA (enriched in unmethylated CpG motifs), acts as a danger signal and triggers cytokine production for a protective and regulated immune response^7,55^. **b** Cytolysis: Under pathological conditions, such as the persistent presence of foreign antigens^104^, “too large to be trapped antigens”, such as fungal hyphae^60,61^, strong adhesion receptor activation^153–155^, presence of monosodium urate (MSU)^88^, or phorbol-myristate-acetate (PMA) stimulation^2,12^, results in an excess of reactive oxygen species (ROS), leading to neutrophil cytolysis. Similarly, excessive increases in intracellular calcium [Ca^2+^]_i_ by ionomycin results in non-apoptotic neutrophil death^12,81^. Certain bacteria are also capable of causing neutrophil cytolysis by releasing pore-forming toxins that can directly cause plasma cell and nuclear membrane permeabilization^156–159^. The externalized cell exudates containing cytoplasmic and nuclear proteins together with damaged nuclear DNA can act as so-called self-antigens (Self-Ag) that are recognized and processed by antigen-presenting cells (APCs). Activated APCs produce pro-inflammatory cytokines and stimulate autoreactive T and B cells, leading to autoantibody production. The circulating autoantibodies such as anti-damaged-DNA/RNA ribonucleoprotein antibody immune complexes (RNP-ICs-Ab) can further activate neutrophils, including NET formation (not shown)^13,73,74^, leading to vicious cycle of chronic inflammation in genetically susceptible individuals^68,74^, causing autoimmune diseases such as systemic lupus erythematous (SLE).

In addition to neutrophils, other granulocyte types can also form extracellular DNA traps, such as eosinophils (eosinophil extracellular traps, EETs)^9,10^ and basophils (basophil extracellular traps, BETs)^11^. Over the last decade, a number of stimuli, microbial and noninfectious stimuli, able to induce extracellular trap formation have been identified^12–16^. It should be noted that there is an ongoing scientific dispute whether NET formation requires cell death or not^17–20^. In this article, we focus on the current evidence of DNA traps under in vivo conditions and discuss their possible role(s) in the disease pathogenesis. These studies suggest that extracellular DNA traps exert effects beyond host defense, including allergic diseases, cancer, vascular diseases, and coagulation. This assumption is best supported by the observation that besides granulocytes, activated T cells, B cells, NK cells, and monocytes are also able to release mtDNA forming extracellular web-like structures, but the latter are devoid of bactericidal proteins. However, these structures containing mtDNA are able to provoke a rapid type I interferon (IFN-I) production in peripheral blood mononuclear cells, suggesting that lymphocytes and monocytes use mtDNA as a rapid signaling molecule to communicate danger^21^. Interestingly, mtDNA being enriched in unmethylated cytosine-phosphate-guanine (CpG) dinucleotide motifs similar to bacterial and viral DNA could provoke IL-10 secretion^21,22^, and transforming growth factor beta (TGF-β) release^23^. IL-10 is known to be an anti-inflammatory cytokine^24^, and TGF-β mediates the suppression of macrophage-directed inflammation, reducing TNF-α release^25^. Combination of anti-inflammatory and regulatory cytokines could dampen the excess inflammation (Fig. 1a).

**Fig. 2:**
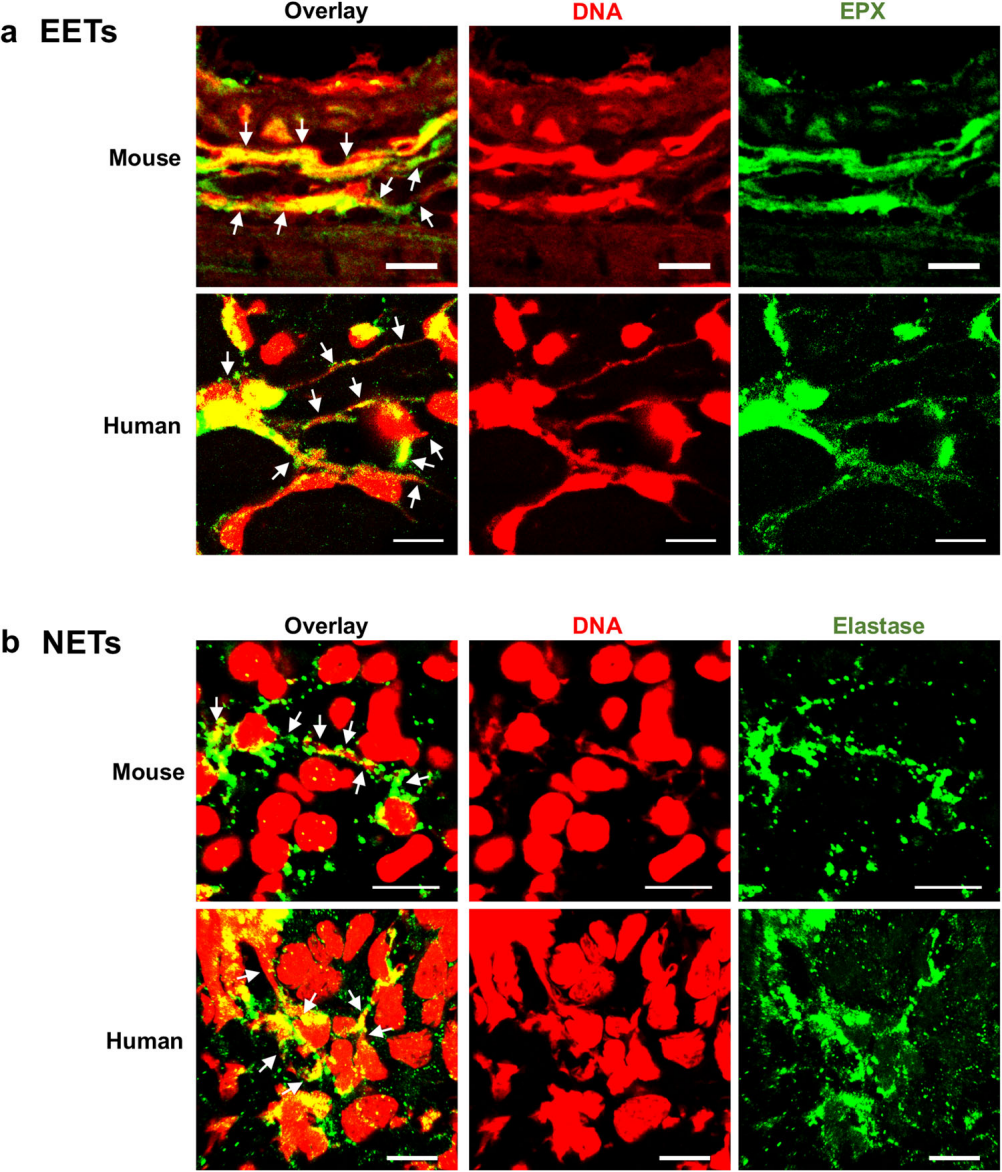
Detection of extracellular traps in tissues. **a** Eosinophil extracellular traps (EETs) in mouse and human tissues. EETs consisting of DNA (red) and eosinophil peroxidase (EPX) (green) are depicted in 12-week-infected *Citrobacter rodentium*-infected mouse colon tissues^55^ (upper panel) and cutaneous *Larva migrans*-infested human skin tissue^26^ (lower panel). **b** Neutrophil extracellular traps in mouse and human tissues. NETs consisting of DNA (red) and neutrophil elastase (green) are shown in overnight infected *Pseudomonas aeruginosa*-mouse lung tissue^7^ (upper panel), and in human lung tissue from patients with bronchial asthma^97^ (lower panel). Extracellular traps are indicated by white arrows. Bars, 10 μm.

The verification of DNA traps in vivo is challenging, as it requires special technical skills^26,27^. Measuring DNA concentrations in the absence or presence of granule proteins is clearly insufficient for concluding the presence of NETs or EETs, but in combination with (immuno)-histological investigations (Fig. 2), these techniques might be used for possible quantification. For example, increased DNA concentrations can occur as a consequence of a lytic granulocyte death (Fig. 1b), but also as a result of tissue damage that is a frequent phenomenon with inflammatory responses. Moreover, in contrast to in vitro conditions, the exact type of stimulus, its dosage and exposure time cannot be assessed under in vivo condition. With these limitations in mind, the reader may understand that the interpretation of the available studies is difficult and it is thus impossible, owing to space limitations, to critically evaluate all cited published findings. We sometimes just reflect the cited work, as it was reported by the original authors. Nevertheless, we often also mention a note of caution.

## The formation of extracellular DNA traps in infectious diseases

The formation of extracellular DNA traps by neutrophils, eosinophils, and basophils, but also lymphocytes, has been observed in various infections of humans, mice, and additional species. Viruses, bacteria, fungi, and parasites have been shown to induce the generation of extracellular DNA traps, which can entrap and even kill the microbes through the action of associated toxic proteins.

### NETs and EETs in bacterial infections

In the bronchial aspirates from patients with acute respiratory infections, bacteria and NETs were visualized using Gram stain and immunostaining. Neutrophils abundantly released NETs, and the NET length that was highest during acute infection and shortened with the recovery, correlated with clinical and laboratory signs of infection, as well as inflammatory cytokine levels in serum^28^. In an animal model of acute respiratory distress syndrome, in which neutrophil infiltration promotes tissue injury and sustained inflammation, the content of cf DNA in bronchoalveolar fluid was significantly higher in mice injected with LPS from *Escherichia coli* as compared with controls, and was decreased by glutamine treatment^29^. It should be noted, however, that in these two studies, NET formation and DNA concentrations were analyzed ex vivo using BAL fluids. To compensate for this shortcoming, lung tissue biopsies should be stained for NET detection in order to confirm the ex vivo data. In addition, cf DNA as well as granule and histone proteins are often quantified as “in vivo NET formation”^30^. Clearly, it is impossible to distinguish whether the released DNA/protein complexes are owing to NET formation or neutrophil death^31,32^, the latter of which was reported to occur under in vivo conditions more than 50 years ago^33,34^.

Genetically modified mice have been used to determine the role of specific proteins for NET formation under in vivo conditions. For instance, the role of peptidylarginine deiminase 4 (PAD4), an enzyme that catalyzes citrullination of histones, has been intensively studied in association with NET formation. Several reports have argued that PAD4 activity is essential for NET formation^14,35–41^, and contrary others disputed that PAD4 is not crucial for NET formation or the antimicrobial defense mechanism in vivo^42–45^. Specifically, NET formation in *Klebsiella pneumonia*-induced pneumonia was investigated showing NET-like structures surrounding *Klebsiella* bacteria at sites of immune infiltration in both *Pad4*^−/−^ and *Pad4*^+/+^ mice. Moreover, both groups showed similar bacterial growth, lung inflammation, and organ injury. In conclusion, these data argue against a major role for PAD4 in NET formation, host defense, or organ injury during pneumonia-derived sepsis^44^.

Cystic fibrosis (CF) is characterized by a chronic inflammation of the airways associated with bacterial colonization. It has been reported that CF neutrophils have a pro-survival phenotype that allows increased NET production, which can in turn induce inflammation^46^. However, it should be noted that this conclusion is based mainly on ex vivo data. Both mucoid and non-mucoid *Pseudomonas aeruginosa* strains were demonstrated to activate neutrophils to generate NETs, a process that is promoted by macrophage migration inhibitory factor (MIF)^47^. Moreover, MIF protein levels in the blood of CF patients were significantly elevated compared with MIF levels in pooled human serum from healthy controls and negatively correlated with lung function^47^. On the other hand, the development of mucoidy (i.e., increased alginate production) is an acquired *Pseudomonas aeruginosa* virulence factor that is closely associated with increased severity of CF. The conversion to a mucoid phenotype coincided with a decline in susceptibility to NETs, raising the possibility that increased alginate production decreases interactions with NETs, or otherwise interferes with killing by NET-associated granule proteins^48^.

*Staphylococcus aureus* may cause serious infections, in particular when complicated by bacteremia and sepsis, and present a common health problem worldwide. In order to unravel the mechanism of organ damage, a mouse model was applied. Intravenous infection with multi-resistant *Staphylococcus aureus* led to a rapid sequestration of the bacteria to the liver, neutrophil recruitment and NET formation within the liver sinusoids, and subsequent liver damage^14^. As neutrophil elastase (NE), a component of NETs, was demonstrated to be enzymatically active and NE staining observed in areas adjacent to focal necrosis, the authors concluded that NET formation largely contributes to liver damage^14^. However, the authors also observed that destroying NETs by DNase treatment only partly reduced tissue injury, leaving some doubt about whether NETs are solely responsible for the immunopathology in this experimental model.

**Fig. 3:**
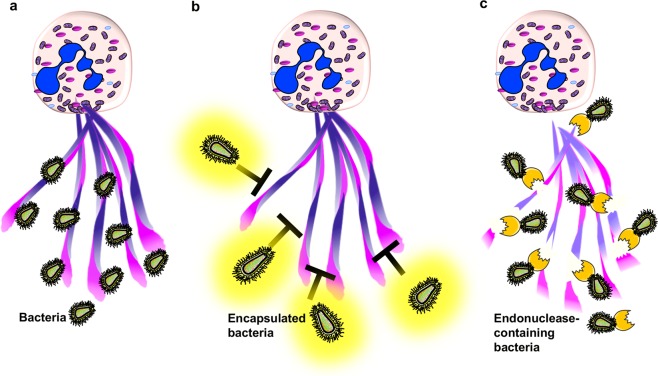
Mechanisms of microorganisms for escape from extracellular traps. **a** Extracellular traps consisting of a DNA scaffold and granule proteins entrap and kill microorganisms^6,7,14,152^. Certain pathogens have developed properties for escaping the physical entrapment by (**b**) encapsulation of bacteria to shield against traps^51^ or (**c**) release of endonucleases to degrade the extracellular DNA scaffold leading to less efficient killing of bacteria^49,50^.

In addition, DNases are expressed by many Gram-positive bacterial pathogens, but their role in virulence is not clear. Expression of a surface endonuclease encoded by *EndA* is a common feature of many pneumococcal strains. *EndA* nuclease allows *pneumococci* to degrade the DNA scaffold of NETs and escape. Escaping NETs promotes spreading of pneumococci from the upper airways to the lungs and from the lungs into the blood stream during pneumonia^49^. Bacterial release of DNase and phosphatases contribute to defense against NET-mediated killing of *Pseudomonas aeruginosa*, highlighting the role of manipulating enzymes in combating NETs by microorganisms^50^. In addition, the pneumococcal polysaccharide capsule protects from NET trapping, but is not required for resistance to NET-mediated killing (Fig. 3)^51^.

In piglets infected with *Streptococcus suis* causing meningitis, NETs that consisted of DNA and associated NE have been detected in the cerebrospinal fluid (CSF)^52^. During pneumococcal meningitis, NETs in the central nervous system have been reported to hinder bacterial clearance. NETs were present in the CSF of patients with pneumococcal meningitis, but absent in other forms of meningitis with neutrophil influx in the CSF^53^. Pneumococci-induced NET formation in the CSF of infected rats could be cleared upon intravenous application of DNase I resulting in a disruption of NETs in the CSF followed by bacterial clearance, suggesting that NETs may contribute to pneumococcal meningitis pathogenesis in vivo^53^.

The formation of NETs has also been observed at cutaneous tick bite sites. Here, NETs have the potential to entrap and kill *Borrelia burgdorferi*, spirochetes causing Lyme disease. NETs have been observed in the upper and deep dermis after 3 and 5 days, respectively, that was not affected by either tick saliva or nucleases derived from these bacteria^54^.

Intestinal eosinophil infiltration and deposition of EETs, which were shown to be able to kill bacteria, were protective against microbial sepsis after cecal ligation in *IL-5*-transgenic, but not wild-type mice^9^. Moreover, infection with *Citrobacter rodentium* resulted in increased frequencies and activation of eosinophils and low bacterial load in the colon of wild-type mice, while eosinophil-deficient mice exhibited a high bacterial load despite the infiltration of neutrophils, Th1, and Th17 cells^55^. In this in vivo model showing that *Citrobacter rodentium* induced formation of EETs with bactericidal effects, and that EETs are present in the colon of infected mice provide further evidence for a role of EETs in controlling bacterial infection^55^.

### NETs in viral, fungal, and parasitic infections

Influenza virus infection creates a highly pro-inflammatory lung environment and neutrophils, which are recruited to the lungs, encounter inflammatory mediators that can trigger NET formation^42^. Upon challenge of mice with lethal doses of influenza virus, NETs were found in infected lungs in the alveoli, and DNA fibers associated with matrix metallopeptidase (MMP)-9 and histones H2B were directed toward the alveolar epithelium and small blood vessels in areas with hemorrhagic lesions, indicating that there exists a contribution by NETs to alveolar–capillary damage^56^. *Respiratory syncytial virus (RSV)* may cause a severe lower respiratory tract disease (LRTD) in young children that is characterized by an extensive neutrophil accumulation in the lungs and occlusion of small airways by DNA-rich mucus plugs. In vitro, NETs were shown to capture *RSV*, and thus prevent its binding to target cells^57^. NET formation was also observed in the airways and lungs of children with severe *RSV*-LTRD. However, their effects in vivo seem double edged as demonstrated in a bovine model of *RSV*-LTRD revealing NET formation either with or without captured viral antigen in the dense plugs that finally occluded the airways^57^. Furthermore, double-stranded DNA (dsDNA) released by NETs recapitulate and promote rhinovirus-induced type-2 allergic immune responses and asthma exacerbation. Thus, NETs and their associated extracellular dsDNA contribute to the pathogenesis and may represent potential therapeutic targets of rhinovirus-induced asthma exacerbations^58^.

*Candida albicans*, a eukaryotic pathogen that is a common cause of fungal infections in humans, particularly in immunocompromised individuals, induces neutrophils to form NETs that capture and kill both hyphal and yeast form cells, for which NET-associated granule proteins are essential^59^. Further, it has been postulated that neutrophil responses, either phagocytosis or NET formation, are regulated by the microbial size independent of fungal surface molecule expression or enzymatic activity in vitro^60,61^. Correspondingly, NET release in the lungs of mice occurred upon exposure to wild-type *Candida albicans* that form both yeast and hyphae, whereas the yeast-locked *hgc1*^Δ^ mutant, that cannot form hyphae, failed to induce NETs, and myeloperoxidase (MPO)-deficient mice able to kill via phagocytosis cleared the *hgc1*^Δ^ yeast-locked strain, but not wild-type *Candida albicans*^61^. These in vivo observations indicate that NETs were not required for the clearance of yeasts, but for controlling hyphae.

In mice infected with larvae of *Strongyloides stercoralis*, an increase of extracellular DNA in the peritoneal exudates was observed 3 h after infection. The authors argued that this observation would be suggestive evidence for NET formation, although direct proof under in vivo conditions was not provided. However, the authors demonstrated in vitro experiments, suggesting that larvae of *Strongyloides stercoralis* induce the release of extracellular DNA forming clot-like structures ensnaring live larvae^62^. In an experimental setting using human neutrophils and macrophages in the presence of complement, both cells collaborated and killed the larvae in a manner requiring NET formation. Interestingly, when mouse cells were used, killing of larvae occurred independent of NET formation^62^.

The protozoan *Toxoplasma gondii* induced NET formation by mouse and human neutrophils with parasite entrapment and killing in vitro. NET formation was also shown in vivo in a mouse intranasal infection model^63^. On the other hand, although infection with *Leishmania mexicana* resulted in NETs entrapping parasites in the tissue of infected mice, in vitro experiments revealed that the induced NETs were unable to kill this protozoan^64^.

NET formation in onchocerciasis (river blindness), a helminth infection, is induced by the release of the bacterial endosymbiont, *Wolbachia*, and not directly by the filarial nematode *Onchocerca volvulus*. The onchocercomata (subcutaneous nodules) of patients contain worms positive for *Wolbachia* and exhibited high numbers of NETs. NETs were found in zones adjacent to the nematode cuticle, whereas nodules derived from patients treated with the anti-*Wolbachia* drugs, doxycycline + ivermectin were depleted for *Wolbachia* and lacked NETs^65^. More recently it was reported that NETs would drive inflammation in malaria by releasing soluble NET components to facilitate parasite sequestration and tissue destruction, and inhibition of NETs as a treatment strategy in vascular infections was recommended^16^.

## The formation of extracellular DNA traps in autoimmune and autoinflammatory diseases

The formation of NETs has been demonstrated in several autoimmune and autoinflammatory diseases. In most studies, NETs appear to be part of the pathological process. Some of the proposed mechanisms are discussed below.

### NETs in systemic lupus erythematosus

Systemic lupus erythematosus (SLE) is considered to be a prototypic systemic autoimmune disease and is characterized by loss of tolerance to self-antigens, abnormal T- and B-cell responses, and autoantibody production^66–70^. Its pathogenesis involves defective clearance of immune complexes and debris containing nucleic acids, excessive innate immune activation involving Toll-like receptors (TLR) and type I interferons, as well as aberrant lymphocyte activation^71^. Upon stimulation with antimicrobial^72^ or anti-ribonucleoprotein (RNP) antibodies^13,73,74^, neutrophils from SLE patients have been shown to release self-DNA associated with antimicrobial peptides able to trigger innate plasmocytoid dendritic cell (pDC) activation via TLR9 to produce IFN-Ι (Fig. 1). The immune complex-mediated glomerulonephritis in SLE has been associated with a non-lytic extrusion of NETs concomitant with clustering of neutrophils within minutes that have immunogenic properties, including enrichment for high mobility group box protein 1 (HMGB1), oxidized mtDNA, and immune complex (ICx) formation^13,73,75^. By applying immunofluorescence staining, NETs composed of mtDNA and MPO have been detected in affected glomeruli^66^. Moreover, the proportion of glomeruli infiltrated by netting neutrophils correlates with lupus nephritis activity. The fact that NETs containing MPO as well as intact neutrophils are present in the dermis and dermal blood vessels of cutaneous lupus lesions suggests that enhanced NET formation occurs in vivo in affected organs of SLE patients^76^. SLE patients were found to develop autoantibodies to both the self-DNA and antimicrobial peptides present in NETs, indicating that these complexes serve as autoantigens to trigger B-cell activation. NETs containing cathelicidin LL-37–DNA complexes can directly trigger human memory B cells and induce the production of anti-neutrophil and anti-LL-37 antibodies in B cells of SLE patients (Fig. 1)^77^.

The timely removal of NETs seems crucial to avoid presentation of self-antigens. For instance, sera of a subset of SLE patients failed to properly clear NETs due to the presence of DNase I inhibitors or and anti-NET antibodies preventing DNase I access to NETs. The resulting impaired NET degradation correlated with renal disease^78^. Moreover, NETs derived from SLE neutrophils exhibit lower ubiquitin concentrations and a different ubiquitinated protein pattern compared with healthy controls^79^. The presence of ubiquitinated MPO in NETs and anti-ubiquitinated MPO antibodies in sera of SLE patients correlated with disease severity, suggesting a role for the ubiquitination status of NETs in the pathogenesis of SLE^79^.

A mitochondrial ROS scavenger was administered to MRL/lpr (lupus-prone mice) resulting in reduced mtDNA release and suppressed lupus-like disease^13^. Metformin, which in vitro decreased the number of mtDNA copies in NETs, significantly reduced the disease activity in SLE patients when given as an add-on therapy compared with conventional therapy alone^66^. Moreover, inhibition of PAD4 was reported to reduce the severity of SLE in an experimental mouse model by reducing histone hypercitrullination and self-nucleosome antibodies^38,80^. Therefore, PAD4 has been suggested as a promising drug target, and novel PAD4 inhibitors have been developed^37–39^. However, a pharmacological approach to inhibit PAD4 in a human serum transfer model of SLE failed to ameliorate end-organ damage and concluded that PAD4 does not appear to be crucial for SLE pathogenesis^45,81^.

### NETs in vasculitis, rheumatoid arthritis, and psoriasis

NETs have also been implicated in the pathogenesis of small vessel vasculitis (SVV) leading to inflammation and destruction of small-sized blood vessels and capillaries. Upon stimulation with anti-neutrophil autoantibodies (ANCAs), neutrophils have been shown to release NETs that contain autoantigens, such as proteinase-3 (PR3) and MPO^82^. On the other hand, NETs with their components mtDNA, PR3, and MPO have been shown to be taken up by myeloid dendritic cells (mDC), significantly inducing the production of anti-neutrophil cytoplasmic autoantibodies (ANCA) and anti-dsDNA autoantibodies in mice immunized with NET-loaded mDC^83^. Therefore, both NET-induced autoimmunity as well as autoantibody-mediated NET formation seem to represent a *vicious circle* in disease pathogenesis. Similarly, drug-induced formation of NETs enriched in NE serving as auto-antigen, together with the release of B-cell activating factor (BAFF) resulting in B-cell activation, and the production of ANCA directed against NE that further enhance NET formation, have been assumed to be pathogenic mechanisms in cocaine and levamisole-associated autoimmunity^84^. A role for NETs in ANCA-associated vasculitis is also suggested by the observation that excessive NET formation correlated with disease exacerbation^85^.

Increased NET formation has also been associated with rheumatoid arthritis (RA). In a mouse model of collagen-induced arthritis (CIA), injecting chloramidine (Cl-amidine), which inhibits PAD4-mediated hypercitrullination, significantly reduced NET release and attenuated clinical disease activity. NETs apparently induced the expansion of Th1 pathogenic cells through maturation of dendritic cells and production of IFN-γ^86^. Therefore, analogous to SLE, NETs from RA patients may exhibit antigenic properties, but may also be recognized by autoantibodies, namely anti-citrullinated antibodies (ACPA)^87^. On the other hand, NETs may promote the resolution of neutrophilic inflammation by degrading cytokines and chemokines and disturbing neutrophil recruitment and activation^88^.

NET-like DNA formations have been detected associated with MPO and IL-17 in the epidermis, particularly in Monro’s microabscesses, and associated with IL-17 and LL-37 in the dermis^89^. However, the observations that the morphology of the NETs varied and most of the neutrophils in Monro’s microabscesses had altered nuclear morphology, suggested that the DNA release was at least partially the result of cell death^17,89^.

### EETs and NETs in bullous pemphigoid

Bullous pemphigoid (BP) is an autoimmune blistering skin disease characterized by an activation of autoreactive B and T cells, the production of pathogenetically relevant autoantibodies directed against the hemidesmosomal proteins BP180 and BP230, and a prominent eosinophil infiltration in the skin^90^. It has been shown that in prebullous lesions, a small subgroup of eosinophils have formed EETs consisting of mtDNA associated with eosinophil granular proteins^26^. Some of these EET were directed against the dermal–epidermal junction, the site where blister formation occurs^26^. Thymic stromal lymphopoietin (TSLP) has been identified as a cytokine-stimulating eosinophils to form EETs, an observation that might be relevant for BP since TSLP is expressed in the epidermis and eosinophils infiltrating BP lesions express the TSLP receptor^10^. In an ex vivo skin model, eosinophils stimulated with IL-5 in the presence of BP serum caused a dermal–epidermal splitting resembling BP. This splitting was significantly decreased upon adding DNase I, suggesting that EET formation plays at least a partial role^90^. These observations make eosinophils interesting targets for therapy^91^. A recent study reported the presence of NETs at sites of blister formation in BP that decreased with time following treatment in patients undergoing remission^92^.

### NETs in autoinflammatory diseases

Autoinflammatory diseases manifest as recurrent fevers, various forms of systemic inflammation or sterile skin, bone, and joint inflammation without prominent fever, but involving myeloid cells that lack the stigmata of classical autoimmune diseases, such as high-titer autoantibodies or antigen-specific T cells^93^. Familial Mediterranean fever (FMF) is characterized by neutrophilia and neutrophil infiltration in affected tissues during inflammatory attacks induced by physical or psychological stress. In vitro, the amounts of NETs consisting of DNA associated with NE and interleukin (IL)-1β, released by PMNs isolated from FMF patients during attack were significantly higher compared with those in remission, when PMNs were resistant to stimuli usually inducing NET formation^94^. Abundant infiltrates of neutrophils forming NETs have been reported in Schnitzler syndrome, defined by recurrent urticarial rash, monoclonal gammopathy, and systemic inflammation^95^. Flares of sterile arthritis with neutrophil infiltrate and the overproduction of IL-1β are the main features of pyogenic arthritis, pyoderma gangrenosum and acne (PAPA) syndrome. NETs have been identified in the skin lesions of a patient with active PAPA syndrome in a milieu characterized by IL-1β, IL-8, and IL-17A expression, but not in skin samples from a patient with no active skin lesions^96^. In vitro, serum of PAPA patients induced NET formation by neutrophils from healthy donors that could be blocked by the IL-1 receptor antagonist anakinra, suggesting that IL-1β contributes to the enhanced NET formation in PAPA^96^.

## The formation of extracellular DNA traps in eosinophilic diseases

The formation of EETs has been demonstrated in several eosinophilic diseases. In most studies, the extent of DNA trap formation correlated with disease severity. On the other hand, bacterial killing by DNA traps appears to be an important innate immune mechanism in case of the presence of a disease-promoting and/or inflammation-induced epithelial barrier defect.

### EETs in asthma and rhinosinusitis

Bronchial asthma is a heterogeneous inflammatory airway disorder that involves eosinophilic and non-eosinophilic, including neutrophilic, phenotypes. In bronchial biopsies, EETs consisting of a mtDNA scaffold co-localizing with MBP were seen in all asthma patients, and their number correlated with the number of eosinophils^97^. A subgroup of patients expressed a high level of neutrophils and NETs, in which the extracellular mtDNA was associated with NE^97^. In an animal model of asthma, the treatment with deoxyribonuclease, which cleaves extracellular DNA, resulted in an improvement of airway resistance and abolished extracellular DNA content in BALF as well as goblet cell hyperplasia^98^. Peripheral blood eosinophils from patients with severe eosinophilic asthma (SEA) may be more activated to produce EETs than those from patients with non-severe asthma (NSA), which further induces inflammation in asthmatic airways^99^. Surfactant protein-D (SP-D), an epithelial cell product of the airways, is a critical immune regulatory molecule with a multimeric structure susceptible to oxidative modifications. We have demonstrated that SP-D directly binds to the eosinophil membrane, inhibits EET formation, and reduces asthma exacerbations^100^. NETs have also been visualized in induced sputum from patients with asthma and COPD, which compared with control sputum, exhibited higher levels of extracellular DNA and other NET components, such as cathelicidin LL-37, alpha-defensin 1–3, NE, IL-1β, and CXCL8 correlating with decreased lung function^101^. In stable COPD patients, extensive NET formation was observed in all sputum samples irrespective of purulence or smoking status^102,103^. Moreover, the presence of NETs is associated with disease severity and microbiota diversity in patients with COPD^104^.

In chronic rhinosinusitis with nasal polyps that is characterized by Th2-biased eosinophilic inflammation, about 8.8% of tissue eosinophils exhibited EETs correlating with IL-5 and periostin tissue levels and colonization with *Staphylococcus aureus* (*S. aureus*)^105^. In an ex vivo human mucosal disease tissue model, transepithelial migration at sites of epithelial defects and massive EET formation of eosinophils to entrap *S. aureus* has been demonstrated^105^. These observations, together with the finding that *S. aureus* can directly induce EET formation, suggested that, in case of epithelial barrier defects, eosinophils are part of the innate immune response for avoiding the invasion by bacteria^10,105^. In a subsequent study, it was demonstrated that EETs, but not NETs, were detected at various degrees in all tissue specimens of nasal polyps or ethmoid tissues obtained by endoscopic sinus surgery for chronic rhinosinusitis. The number of EETs correlated with that of tissue eosinophils, blood eosinophilia, severity and decreased olfactory function, regardless of the presence of nasal polyps, concomitant asthma or atopy^106^.

### EETs in acute dermatitis responses and eosinophilic esophagitis

When specimens of various eosinophilic skin diseases were examined, EETs could be detected in atopy patch test-induced lesions, but not in biopsies from atopic dermatitis or in positive patch test reactions of patients with allergic contact dermatitis, suggesting that EET formation occurs as an acute response to allergens^26^. Induced lesions in a patient with bullous delayed-pressure urticaria showed a marked infiltration of eosinophils, of which >80% formed extracellular DNA traps, suggesting a pathogenic role of eosinophils and EETs^107^.

In eosinophilic esophagitis, EET formation occurred frequently, as it was detected in all tissue samples analyzed, and correlated with the number of tissue eosinophils^108^. Moreover, there was evidence for epithelial barrier defects, e.g., decreased filaggrin and protease inhibitor LEKTI expression, while LEKTI inversely correlated with the number of EETs^108^. These findings imply a role for eosinophils in protecting the host against invading pathogens in case of a disrupted epithelial barrier by generating EETs and thus establishing a second barrier.

## The formation of extracellular DNA traps in cardiovascular diseases

Several non-microbial stimuli for NETs have been described including cholesterol, which can trigger and amplify sterile inflammation^109^. Such stimuli likely play a role in NET formation associated with cardiovascular diseases.

### NETs in atherosclerosis

The presence of NETs has been reported in mice and human atherosclerotic lesions. Using a two photon microscopic intravital approach, luminally adhering neutrophils releasing DNA in apolipoprotein-deficient (*Apoe*^−/−^) mice fed a high-fat diet for 4–6 weeks were observed, whereas no neutrophil adhesion and hence no NET release were detected in mice receiving chow diet^110^. Together with NETs, associated antimicrobial peptide Cramp/LL-37 and plasmacytoid dendritic cells (pDC) have been identified in atherosclerotic lesions. Cramp/DNA complexes can stimulate pDC to produce IFN-α, that in turn promotes atherosclerotic plaque growth that is associated with enhanced anti-dsDNA antibody titers^111^. In order to study atherosclerosis in the absence of NETs, *Apoe*^−/−^ mice lacking NE and PR3 (*Apoe*^−/−^*Elane*^−/−^*Prtn3*^−/−^) were employed and revealed reduced lesions size after 8 weeks of high-fat-diet feeding^112^.

Inhibition of PAD4 by Cl-amidine resulted in decreased atherosclerotic lesion size and delayed carotid artery thrombosis in the *Apoe*^−/−^ mouse model^80^. The authors also showed that PAD4 inhibition mitigates arterial IFN-I responses, and reduces the number of netting neutrophils that infiltrate the media and adventitia of atheromatous lesions. Although the authors concluded a causative role for NETs in the development of murine atherosclerosis^80^, it should be again noted that a requirement for PAD4 for NET formation, and its correlation with diseases, is in dispute^17,19,42–45^. In fact, more recently, selective genetic deficiency of *Pad4* in bone marrow-derived cells exhibited no differences in the formation and progression of atheromatous plaques compared with control mice^113^.

In mice, cholesterol accumulation in myeloid cells activates the NOD-like receptor protein (NLRP3) inflammasome which enhances neutrophil accumulation and NETs formation in atherosclerotic plaques^114^. Furthermore, activated platelets were determined as physiological stimulus that via P-selectin induce NET formation^115^.

Do these findings obtained in murine models correlate with human disease? In patients with coronary artery disease (CAD) proven by CT-angiography markers of cell death, NET formation in plasma was measured, revealing significantly elevated levels of dsDNA, nucleosomes, and MPO–DNA complexes^116^. Moreover, a high plasma nucleosome level was identified as an independent risk factor for severe coronary stenosis, and the level of MPO–DNA complexes predicted the number of atherosclerotic vessels^116^. In patients with stable coronary artery disease who had high dsDNA plasma levels, the risk of experiencing a clinical endpoint defined as unstable angina, non-hemorrhagic stroke, myocardial infarction, or death was significantly increased after 2 years^117^. However, it still remains to be established whether circulating extracellular DNA in patients indeed reflects the extent of NET formation. Nevertheless, host DNases prevented vascular occlusion by NETs^118^.

### NETs in atherothrombosis

Progression of atherosclerotic lesions can eventually lead to the destabilization of plaque with subsequent intraluminal atherothrombosis resulting in acute coronary syndrome or ischemic stroke, depending on the plaque location. For initiating arterial thrombosis in injured vessels, the interaction of neutrophils with endothelial cells is a critical step preceding platelet accumulation^119^. Activated lesional smooth muscle cells (SMCs) may attract neutrophils that undergo neutrophil death (not NET formation), releasing nuclear proteins, including histone H4, which induce lytic cell death of smooth muscle cells causing the destabilization of plaques. Neutralization of histone H4 led to a plaque stabilization, whereas blocking histone H2A or disrupting DNA structures by DNase had no effect on smooth muscle cells^120^. A significant decrease in infarction size and reduction of citrullinated histone 3 in infarcted tissue were demonstrated upon blocking of CCL5 and CXCL4 in mice, what was believed to be due to a reduced neutrophil recruitment^121^. In a model of myocardial infarction, *Pad4*^−/−^ mice were significantly protected from, whereas mice without PAD4 deficiency had myocardial injury with the presence of citrullinated histone H3 at the site of injury and increased plasma levels for nucleosomes^122^. Our interpretation of these findings is that a lytic neutrophil death might be prevented in the absence of PAD4. Therefore, a lytic neutrophil death and not of NET formation seems to be important for atherothrombosis.

On the other hand, studies investigating atherothrombosis in humans revealed evidence for the presence of NETs in thrombi after endarterectomy. NETs were identified by immunohistochemical staining, e.g., with anti-MPO and anti-NE antibodies, in fresh and lytic, but not in organized thrombi. The presence of NETs was evident in the thrombi and hemorrhages as well as at the thrombus-plaque interface and in perivascular tissue adjacent to complicated, but not intact plaques^123^. In coronary thrombi, the NET burden was found to correlate with the infarct size and was negatively related to plasma DNase activity^124^. In addition to neutrophils, also macrophages, eosinophils, and mast cells have been observed to form DNA traps in coronary thrombi^125^.

### NETs in venous thrombosis and thromboembolism

Deep vein thrombosis (DVT) has been linked to neutrophil activation and release of NETs based on studies investigating the pathogenic role of NETs in the pathogenesis of venous thromboembolism (VT) using genetically modified mice, various large animal models and human material assessing plasma markers or thrombi species^126^.

In a mouse DVT model applying intravital microscopy, neutrophils and monocytes, crawling along and adhering to the venous endothelium, have been shown to provide the initiating stimulus for DVT^127^. A cooperative signaling of P-selectin glycoprotein ligand (PSGL)-1 and CXCR2 in neutrophils increases their adhesion and enhances murine venous thrombosis through NET formation in flow-restricted veins^128^. Furthermore, platelet-derived HMGB1 as well as NK cell-dependent IFN-γ production were also reported to stimulate NET formation and thus contribute to the development of thrombi in deep vein thrombosis^129,130^. Thrombus-resident neutrophils bind factor XII and activate it through the release of NETs, whereas disintegration of NETs protected against DVT amplification^127^. In mice with DVT induced by flow restriction in the inferior vena cava, extracellular DNA was increased in plasma, and citrullinated histone H3 associated with neutrophils in venous thrombi^131^. Both the DNA scaffold and histones were shown to contribute to DVT^131^.

The analysis of 16 thrombi from patients with VTE demonstrated that NETs, defined as extracellular diffuse citrullinated histone 3 areas associated with MPO and DNA, were predominantly detected during the phase of thrombus organization, while NETs were rare in unorganized and organized thrombi^122^. As biomarkers of DVT, extracellular DNA and MPO have been tested. Plasma DNA levels are elevated in patients with DVT correlating with C-reactive protein, D-dimer, von Willebrand factor, and MPO^132^. Moreover, in elderly patients, levels of circulating extracellular DNA correlated with the extent of venous thromboembolism, inflammation as assessed by C-reactive protein and leukocytosis, and predicted mortality^133^. Recently, flow cytometric detection of MPO/citrullinated histone 3-positive neutrophils and serum dsDNA have been proposed for biomarker assessment^30^. However, as mentioned earlier, these biomarkers are not suitable for the definition of NET formation and can also not be used for mechanistic studies.

To date, clinical trials investigating whether targeting NETs prevents or treats venous thrombosis in humans are lacking. However, some established therapies of venous thromboembolism may affect extracellular DNA traps^126^. For instance, heparin was shown to dismantle the DNA scaffold and remove platelet aggregates from NETs^134^. Acetylsalicylic acid prevents NET formation in vitro by reducing the phosphorylation of the NF-κB p65 subunit^135^. In mice, acetylsalicylic acid inhibited tissue factor activity and NET formation followed by a marked reduction of thrombus size^136^. Clopidogrel, by decreasing P-selectin expression in platelets, blocks the interaction with neutrophils and thus NET formation^137^.

## The formation of extracellular DNA traps in cancer

**Fig. 4:**
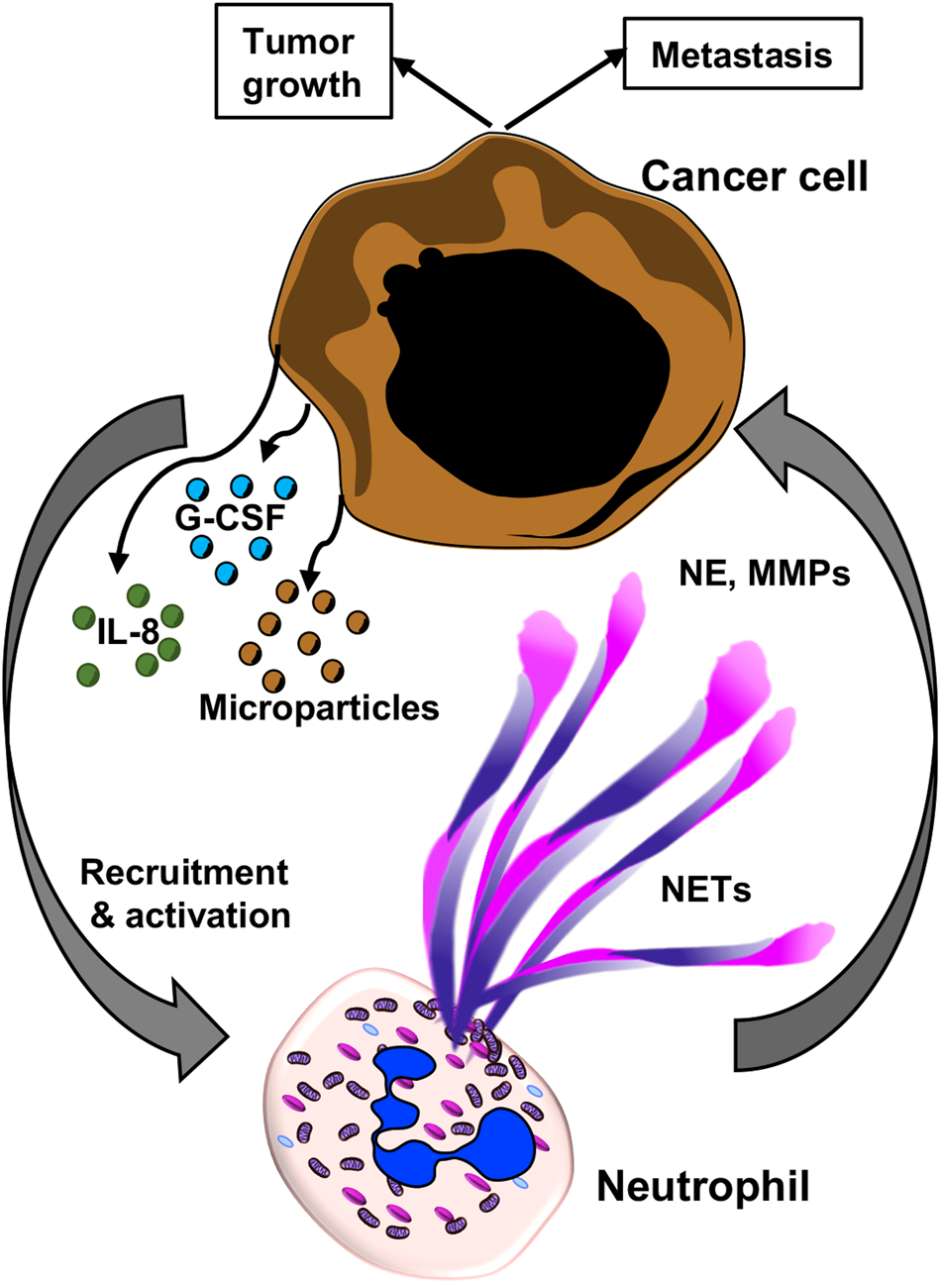
Role of extracellular traps in cancer and metastasis. Interactions between tumor cells and neutrophils determines the outcome of tumor growth, progression, and metastasis. Soluble factors and inflammatory mediators such as IL-8 and granulocyte colony-stimulating factor (G-CSF), as well as microparticles released from tumor cells prime and activate neutrophils to form NETs. NETs-associated granule proteins can contribute to tumor metastasis by releasing proteases such as matrix metalloproteases (MMPs) and neutrophil elastase (NE) that allows tumor cells to move out of the primary niche and to migrate to other organs^143,151,160^.

Hypothetically, NETs could have anti-tumorigenic effects, for example by actual killing of tumor cells or activating the immune system. However, there is more experimental evidence for a pro-tumorigenic activity of NETs (Fig. 4). First evidence of NET formation by tumor-associated neutrophils came from a histopathological analysis of diagnostic biopsies from Ewing sarcoma. Out of eight tissue samples, tumor-associated neutrophils were detected in six and NETs in two patients. In this study, NET formation was associated with relapsing and metastatic disease despite chemotherapy^138^.

In several mouse models, NET formation has been demonstrated to be associated with tumor growth and/or metastasis (Fig. 4), e.g., NETs have been reported to enhance the growth of melanoma^139^, gastric cancer^140^, and hepatocellular cancer^141^. Moreover, cancer cells themselves are able to stimulate neutrophils to form NETs that facilitate cancer cell migration and invasion as shown in vitro and in vivo, indicating that they make use of a physiological host defense mechanism process to promote metastasis (Fig. 4)^142^. Mechanisms by which cancer cells may stimulate neutrophils to form NETs are the production of IL-8 and the release of exosomes requiring additional priming with granulocyte colony stimulating factor (G-CSF) (Fig. 4)^142,143^. More recently, it was reported that anaplastic thyroid cancer (ATC) cells induce the release of mitochondrial extracellular DNA traps by viable neutrophils. ATC conditioned medium (CM)–primed neutrophils promoted ATC cell proliferation in a NET-dependent manner^144^. Furthermore, tumor cells have been demonstrated to produce IL-8, attracting myeloid-derived suppressor cells (MDSC) and activating granulocytic MDSC to extrude DNA nets^145^. In addition, by applying intravital microscopy, a significant increase in the in vivo hepatic adhesion of intrasplenically injected lung or colon cancer cells was observed in the presence of NETs when compared with animals, in which NET formation had been prevented^146^. Although tumor-infiltrating neutrophils were rare in tissue specimens of epithelial ovarian cancer, both intact neutrophils and NETs were observed in tumors from 4 of 5 patients^147^. Moreover, in ascites supernatants of patients with advanced tumors, high mtDNA, and NE levels were found that correlated with reduced progression-free survival^148^.

In order to investigate the association of severe postsurgical infection and adverse oncologic outcome, a murine model of infection using cecal ligation and puncture was applied, demonstrating microvascular NET deposition and trapping of circulating lung carcinoma cells that was associated with increased formation of hepatic metastases following tumor cell injection^149^. Similarly, surgical stress employing liver ischemia reperfusion resulted in an increase in NET formation with subsequent development and progression of metastatic disease, while pretreatment of mice with topical DNase application or a PAD4 inhibitor abrogated these effects^150^. Consistent with the observation in mice, increased postoperative NET formation inversely correlated with disease-free survival times in a cohort of patients undergoing attempted curative liver resection for metastatic colorectal cancer^150^. It should be noted, however, these authors measured serum levels of MPO–DNA complexes and did not directly analyze NET formation. Therefore, it could very well be that metastasis was associated with neutrophil cell death and not with NET formation.

Cancer cells remain dormant for a long time. In a mouse model of lung inflammation induced by either tobacco smoke or lipopolysaccharide, NET formation was shown to be essential for awakening dormant cancer cells. NETs facilitated the contact of associated proteases NE and MMP9 with their substrate laminin (Fig. 4), resulting in the cleavage of laminin and revealing an epitope that activated integrin-mediated signaling and thus proliferation of dormant cancer cells^151^.

## Conclusion

A beneficial role of NETs and EETs is undisputable, as several studies have demonstrated its antimicrobial activities. On the other hand, they might be able to trigger additional immune responses. Based on recently published work, extracellular microbial killing and phagocytosis act in synergy to effectively remove intracellular and extracellular pathogens regardless of their sizes. A simple biomarker that reflects NET and/or EET formation is currently not available. However, extracellular DNA traps can be detected in inflamed tissues using fluorescence/immunofluorescence techniques. The mechanism of extracellular DNA trap formation remains highly controversial that includes questions regarding the requirement of cell death, the source of DNA, as well as intracellular signaling pathways.
